# Genome sequence of adherent-invasive *Escherichia coli *and comparative genomic analysis with other *E. coli *pathotypes

**DOI:** 10.1186/1471-2164-11-667

**Published:** 2010-11-25

**Authors:** John HE Nash, Andre Villegas, Andrew M Kropinski, Renan Aguilar-Valenzuela, Paulina Konczy, Mariola Mascarenhas, Kim Ziebell, Alfredo G Torres, Mohamed A Karmali, Brian K Coombes

**Affiliations:** 1 Office of Biotechnology, Genomics and Population Health, Public Health Agency of Canada, 180 Queen Street West, Toronto, Ontario M5V 3L7, Canada; 2 Laboratory for Foodborne Zoonoses, Public Health Agency of Canada, 110 Stone Road West, Guelph, Ontario N1G 3W4, Canada; 3 Department of Microbiology and Immunology, University of Texas Medical Branch, Galveston, Texas, 77555-1070, USA; 4 Michael G. DeGroote Institute for Infectious Disease Research and the Department of Biochemistry and Biomedical Sciences, McMaster University, 1200 Main Street West, Hamilton, Ontario L8N 3Z5, Canada

## Abstract

**Background:**

Adherent and invasive *Escherichia coli *(AIEC) are commonly found in ileal lesions of Crohn's Disease (CD) patients, where they adhere to intestinal epithelial cells and invade into and survive in epithelial cells and macrophages, thereby gaining access to a typically restricted host niche. Colonization leads to strong inflammatory responses in the gut suggesting that AIEC could play a role in CD immunopathology. Despite extensive investigation, the genetic determinants accounting for the AIEC phenotype remain poorly defined. To address this, we present the complete genome sequence of an AIEC, revealing the genetic blueprint for this disease-associated *E. coli *pathotype.

**Results:**

We sequenced the complete genome of *E. coli *NRG857c (O83:H1), a clinical isolate of AIEC from the ileum of a Crohn's Disease patient. Our sequence data confirmed a phylogenetic linkage between AIEC and extraintestinal pathogenic *E. coli *causing urinary tract infections and neonatal meningitis. The comparison of the NRG857c AIEC genome with other pathogenic and commensal *E. coli *allowed for the identification of unique genetic features of the AIEC pathotype, including 41 genomic islands, and unique genes that are found only in strains exhibiting the adherent and invasive phenotype.

**Conclusions:**

Up to now, the virulence-like features associated with AIEC are detectable only phenotypically. AIEC genome sequence data will facilitate the identification of genetic determinants implicated in invasion and intracellular growth, as well as enable functional genomic studies of AIEC gene expression during health and disease.

## Background

Crohn's Disease (CD) is a chronic inflammatory bowel disease of the intestinal tract characterized by a strong activation of the intestinal immune system. A complex interaction of genetic, immunologic, and environmental factors contribute to the immunopathology of CD but despite intensive investigation over the last half-century, a unifying etiology of inflammatory bowel diseases (IBD) has not been uncovered [1,2]. Abundant clinical and experimental data implicate luminal bacteria or bacterial products in both the initiation and perpetuation of chronic intestinal inflammation [2-4]. Some pathological manifestations observed in CD, including ulcers of the mucosa, mural abscesses and macrophage recruitment and activation, also occur in well-recognized infectious diseases caused by *Shigella*, *Salmonella *and *Yersinia*, in which invasion into mucosal epithelial cells is an important virulence trait [3]. However, a growing body of evidence indicates that the balance between host defence responses and the commensal microbiota plays a key role in the pathogenesis of IBD [2]. Patients with CD display an increased number of coliforms in their feces, particularly during periods of active disease [5] and *E. coli *antigens are found in most intestinal resection specimens from these patients [6]. Furthermore, it has been shown that early and chronic ileal lesions of CD patients harbour high levels of *E. coli *that might participate in disease pathogenesis [7-11]. *E. coli *strains isolated from the ileal lesions of CD patients can exhibit adherent and invasive capabilities in both gastrointestinal epithelial cells and macrophages [10,12], a phenotype that was the basis for a new pathogenic group called adherent and invasive *E. coli *(AIEC) [12,13]. AIEC are enriched in ileal lesions in human CD [7] and are associated with expression of proinflammatory cytokines and inflammation in mice expressing human carcinoembryonic antigen-related cell adhesion molecule (CEACAM) receptors [14]. The predominance of AIEC in human CD patients, in conjunction with a growing body of biological and animal model data [15] has generated intense interest into the possible role of AIEC in the initiation or maintenance of chronic inflammation associated with CD.

We previously reported on a clinical AIEC isolate with serotype O83:H1 (strain NRG857c) that was isolated from the terminal ileum of a patient with CD [16]. NRG857c belongs to the same serogroup as the historical AIEC isolate called LF82 first described over a decade ago [10] for which much of the experimental data on AIEC phenotypes have been documented. AIEC do not harbour common virulence factors found in various other pathogenic *E. coli*, and so the genetic basis for their invasive phenotype, proinflammatory nature and association with CD are not fully understood. Here, we report the complete genome sequence of AIEC NRG857c that includes a 150-kb plasmid. We found that AIEC are closely related to a group of extraintestinal pathogenic *E. coli *(ExPEC) associated with urinary tract infections and neonatal meningitis, a finding that confirms and extends previous work [17]. The comparison of this genome with other ExPEC, enteropathogenic *E. coli*, AIEC LF82, and commensal *E. coli *facilitated the identification of 41 high-confidence genomic islands and 66 genes unique to *E. coli *displaying the adherent and invasive phenotype.

## Results and Discussion

### Genome sequencing and gap closure

AIEC strain NRG857c was shotgun sequenced to 40-fold coverage using pyrosequencing. Assembly of the raw sequence data generated 48 contiguous regions (contigs) greater than 2-kb with a total size of 4.84-Mb. Contigs were assembled by aligning the larger contigs to an optical restriction map using MapSolver and by BLASTX analysis of contigs ends. The majority of gaps between contigs were identified because contigs ends were syntenic with single-copy genes in previously sequenced *E. coli *genomes. PCR primers were designed to amplify across these gaps followed by sequencing to generate "super-contigs" (see Additional File 1, Figure S1). Final gap closure was achieved after incorporation of sequence data for the seven ribosomal RNA operons. Plasmid contigs were identified by BLASTX analysis. Gap closure for the plasmid was done using BLASTN analysis of the terminal sequences from which PCR primers were designed. Amplification and sequencing of these regions resulted in the assembly, but not closure, of a single plasmid contig.

### General features of the NRG857c AIEC genome

**Figure 1:**
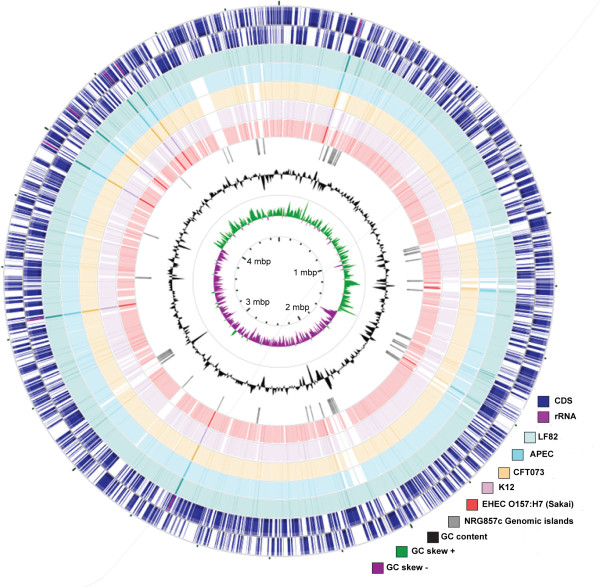
**Comparative genome atlas of NRG857c**. The chromosome of NRG857c (two outermost rings are CDS on forward and reverse strand) was compared with those of selected *E. coli *strains, starting from the outer layer LF82 (AIEC; pale green), APEC-O1 (APEC; blue), CFT073 (UPEC; yellow), MG1655 (K12/commensal; purple) and enterohemorrhagic *E. coli *O157:H7 Sakai (EHEC, red). Genomic islands were plotted on the NRG857c chromosome (grey blocks). The G+C content and G/C skew are also plotted as indicated.

The chromosome of NRG857c is 4,747,819 bp (50.68% G + C content), encoding 4,431 genes (Figure 1, Table 1). The plasmid is 147,060 bp (50.92 G+C content) and encodes 155 genes (Table 1). The sequence of both the NRG857c chromosome and plasmid has been deposited in GenBank [GenBank: CP001855, GenBank: CP001856].

**Table 1:** General features of NRG857c genome and other E. coli strains

				Chromosome						Plasmid(s)	
				
Strain	Serotype	Pathotype	Phylogroup	**Accession No**.	Size (kb)	Total CDS	CDS density (%)	G+C (%)	Total tRNAs	**Accession No**.	Size (kb)
NRG857c	O83:H1	AIEC	B2	CP001855	4,748	4,431	88.2	50.7	84	CP001856	147
LF82	O83:H1	AIEC	B2	CU651637	4,773	4,312	87.7	50.7	84	CU638872	108
E2348/69	O127:H6	EPEC	B2	FM180568	4,965	4,703	88.2	50.6	92	FM180569 FM180570	97, 6
UTI89		UPEC	B2	CP000243	5,065	5,066	91.1	50.6	88	CP000244	114
CFT073	O6:K2:H1	UPEC	B2	AE014075	5,231	5,473	91.9	50.5	89		
536	O6:K15:H31	UPEC	B2	CP000247	4,938	4685	88.7	50.5	81		
APEC-O1	O1:K1:H7	APEC	B2	CP000468	5,082	4,467	87.5	50.6	94	DQ381420 DQ517526	241, 174, 105, 46
O157 Sakai	O157:H7	EHEC	E	BA000007	5,498	5,361	88.1	50.5	105	AB011548 AB011549	92, 3
MG1655 (K-12)	OR:H48:K-	Commensal	A	U00096	4,639	4,294	89.0	50.8	88		
HS	O9	Commensal	A	CP000802	4,643	4,478	88.7	50.8	88		
E24377A	O139:H28	ETEC	B1	CP000800	4,979	4,873	88.6	50.6	91	CP000795- CP000799 CP000801	79, 74, 70, 34, 6, 5

### Phylogenetic position of NRG857c

**Figure 2:**
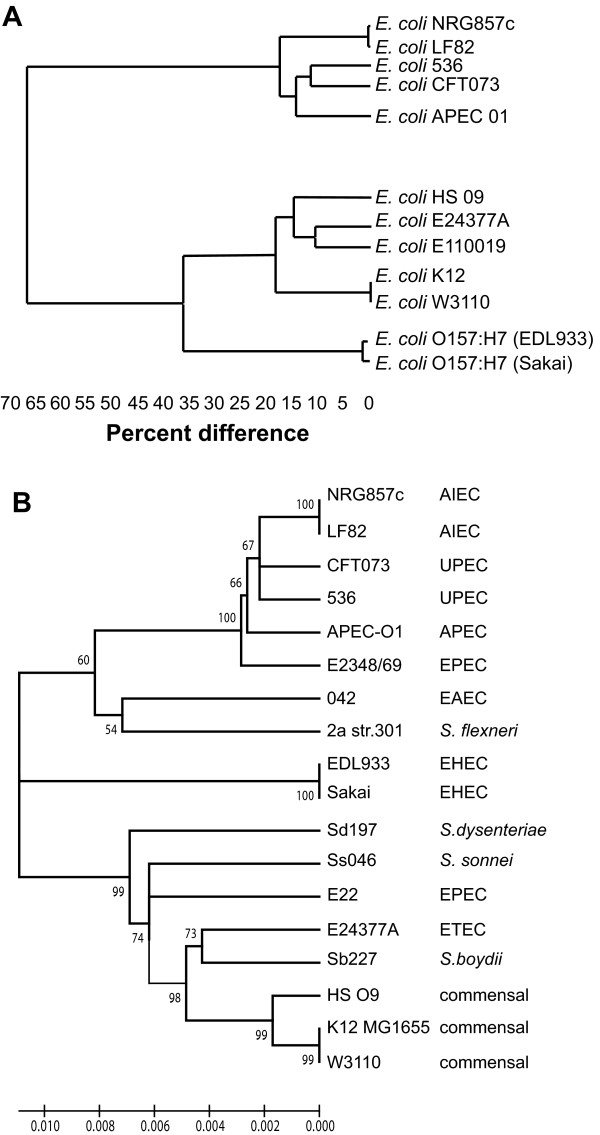
**Phylogenetic analysis of NRG857c compared with representative strains of other enteric bacteria**. **(A) **A phylogenetic tree based on the unweighted pair group method with arithmetic mean was constructed from the optimal map data and *in silico *Nco*I *restriction digests of other enteric bacterial chromosomes. **(B) **MLST-based analysis of NRG857c with other enteric bacteria was performed as described in the Methods and sequence data was used to construct a phylogenetic tree. Numbers on the tree branches represent bootstrap support from 1000 bootstrap replicates with a minimum cut-off of 65%. Accession numbers for gene sequences can be found in Additional File 2, Table S1.

The phylogeny of AIEC NRG857c was resolved in two ways. First, a phylogenetic tree based on the optical map data was constructed using the unweighted pair group method with arithmetic mean (UPGMA) along with the *in silico *derived Nco*I *fragments for other sequenced *E. coli *strains (Figure 2A). The second method involved multi-locus sequence typing (MLST) with seven housekeeping genes as described previously [18] (Figure 2B; Additional File 2, Table S1), followed by comparison to sequences from other strains [19]. In both analyses NRG857c clustered with avian pathogenic *E. coli *(APEC-O1), and the uropathogenic *E. coli *isolates 536 and CFT073. Also in this group was LF82, another AIEC strain of the same serotype as NRG857c (O83:H1) whose genome sequence was retrieved from Genoscope (http://www.genoscope.cns.fr see note added in revision). LF82 shows high sequence similarity to our strain as analyzed by MapSolver (Additional File 3, Figure S2), by BLASTN analysis (Figure 1), and by phylogenetic analysis (Figure 2).

A general comparison of the total genome content of NRG857c with several other *E. coli *pathotypes is shown in Table 1. The majority of human ExPEC belong to phylogenetic group B2 and are categorized based on their clinical spectrum of disease, including urinary tract infections (UPEC) and neonatal meningitis (NMEC) [20-23]. AIEC strains cluster genetically with ExPEC and share some of their phenotypic traits including the ability to colonize mucosal epithelial cells, invade eukaryotic host cells, and to induce inflammatory responses in host animals [24,25]. Although the prototype EPEC strain E2348/69 (serotype O127:H6) and other EPEC strains belong to the same phylogenetic group as the ExPEC strains [26], they are not generally considered to be invasive organisms. However, recent data suggests that at least two type III secreted proteins (EspT and EspF) can facilitate EPEC invasion into non-phagocytic cells and may define a new category of invasive EPEC [27,28].

### Genomic islands and unique sequences associated with AIEC

**Figure 3:**
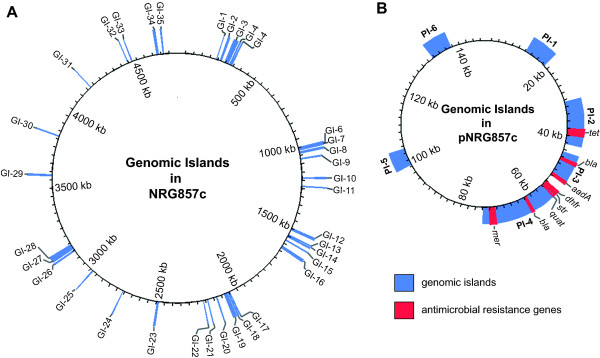
**Genomic islands in NRG857c**. Genomic islands in the NRG857c chromosome **(A) **and plasmid **(B) **were predicted using stringent bioinformatics criteria as described in the Methods. Genomic islands are plotted to scale in blue and labelled clockwise on the genome maps. On the plasmid, genes involved in antimicrobial resistance are indicated in red.

Genomic islands (GI) comprise a horizontally acquired flexible gene pool that is a major driver in evolution and niche specialization of pathogenic bacteria [29]. Recent computational methods that take advantage of genetic signatures indicative of horizontal gene transfer enable the high-confidence prediction of GIs in annotated bacterial genomes [30]. To identify putative genomic islands in NRG857c, we used IslandViewer, which uses three independent methods for island prediction, IslandPick, IslandPath-DIMOB and SIGI-HMM. Using the methods and established thresholds described previously [31], we identified 35 genomic islands (GI-1 to GI-35) on the NRG857c chromosome ranging from 4 to 25-kb, with G+C content differing significantly from genome mean and with poor conservation among the other non-AIEC pathotypes shown in Figure 1 (see Additional File 4, Table S2 for full list of genomic islands and gene content analysis). We limited our comparative analysis here to the strains most related to NRG857c and to two well-described *E. coli *strains of commensal and pathogenic nature. The conservation of these 35 islands between NRG8578c and LF82 was high, suggesting that they may encode traits unique to the adherent and invasive phenotype. Five of the genomic islands (GI-6, -7, -8, -10 and -16) code for defective prophages, three (GI-14, -22, -29) are fimbrial islands, and three (GI-20, -26 and -30) appear to be involved in lipopolysaccharide or capsular polysaccharide biosynthesis. GI-23 is noteworthy because it encodes an EmrKY-TolC multidrug resistance efflux pump and the sensor kinase, EvgA, involved in acid resistance and multidrug resistance in *E. coli *[32]. GI-15 and GI-19 appear to be metabolic islands involved in the transport and metabolism of various sugars. An additional six genomic islands were identified on the large plasmid (PI-1 to PI-6 in Figure 3) (see Additional File 4, Table S2 for full list of plasmid islands and gene content analysis).

To date, restriction profiles or other biased analyses such as pulse field gel electrophoresis (PFGE), MLST or typing for known virulence genes common to intestinal pathogenic *E. coli *have failed to uncover unique genetic determinants implicated in the AIEC phenotype [17]. To begin to identify single genetic determinants unique to AIEC, we carried out whole-genome comparisons between NRG857c, LF82, and 29 other non-AIEC genomes of *E. coli*. NRG857c and LF82 show considerable sequence similarity and synteny (Additional File 3, Figure S2) with 46 chromosomal genes unique to NRG857c and 10 chromosomal genes unique to LF82 (see Additional File 5, Table S3 for full list of genes unique to AIEC). The large plasmids from NRG857c and LF82 show almost no conservation between them (see below), suggesting that they have different ancestry.

Panseq, a Web-based tool designed to analyse the "pan-genome" of closely-related genome sequences, was used to identify genes common to AIEC strains NRG857c and LF82, but absent in other members of this phylogenetic cluster (i.e. APEC-O1, 536, and CFT073). We programmed Panseq to find unique sequences of at least 2-kb present in NRG857c and LF82 but absent in APEC-O1, 536 and CFT073. In this analysis, we found 21 sequences with a combined length of 155-kb that are unique to AIEC strains. Several of these sequences code for prophage elements including a 19.7-kb region encoding the morphogenesis and packaging modules of a P22-like prophage (NRG857_04720 - NRG857_04815). A second interesting region of 47.2-kb extends, with one interruption, from NRG857_09990 to NRG857_10240 and codes for several proteins involved in intermediary metabolism including transport of propanol/propanediol and galactitol. BLASTN analysis of this region revealed two sub-regions, one 20.3-kb and the other 4.4-kb, which are not found in the complete genome sequence of any other *E. coli *strain. The latter region shows 71% sequence coverage to a region from the complete genome of *Citrobacter rodentium *ICC168, while approximately half of the longer sequence is also found in an uncharacterized *E. coli *strain ATCC 8739. This 10.7-kb region has no nucleotide similarity with any other fully sequenced bacterium. BLASTX revealed similarity in this region to two hypothetical *Vibrio coralliilyticus *ATCC BAA-450 proteins [GenBank: ZP_05883689, GenBank: ZP_05883688] adjacent to orthologs in *Burkholderia cenocepacia *HI2424 [GenBank: YP_833853, GenBank: YP_833854], which are described as hypothetical proteins.

### Plasmid analysis

**Figure 4:**
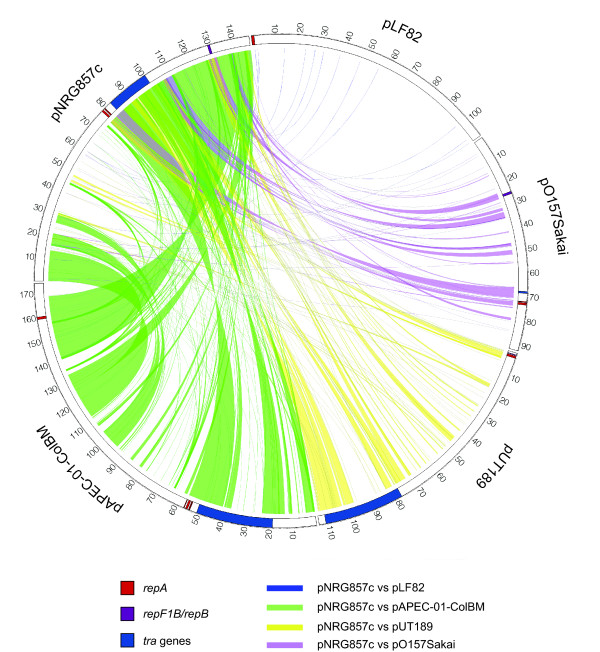
**Gene content analysis of plasmid pNRG857c and comparison to representative strains of other *E. coli***. BLASTN analysis was performed between each CDS in plasmid pNRG857c against each CDS in pLF82, pO157Sakai, pUTI89, and pAPEC-O1-ColBM. Genes in pNRG857c with orthologs in the other plasmids, defined as >85% identity over entire length of the gene, are connected with a coloured line.

The 150-kb plasmid in NRG857c is different from the plasmid found in LF82. Whereas plasmid pNRG857c shows significant regions of identity to plasmids in other seropathotypes of *E. coli*, the 110-kb plasmid of strain LF82 (pLF82) has very little similarity to pNRG857c or pAPEC-O1 (APEC-O1), pColBM (APEC-O103), pUTI189 (UPEC UTI189) and pO157 Sakai (EHEC O157:H7) (Figure 4). The extrachromosomal plasmid in NRG857c is a antimicrobial resistance plasmid with a suite of genes encoding resistance to aminoglycosides, β-lactams, chloramphenicol, mercury, quaternary ammonium salts, sulfonamides, tetracycline, and trimethoprim, several of which appear to be enclosed as transposon blocks. The plasmid may be capable of conjugal transfer as it encodes several *tra *genes, although we have not experimentally tested this. In addition, there are genes for colicins M and V production and immunity. The antibiotic resistance genes are clustered in three regions of the plasmid in PI-2, PI-3 and PI-4 (Figure 3B). The mercury resistance cassette is identical to IS5075 found in IncA/C2 plasmids pRYC103T24 [GenBank: GQ293500.1], pLEW517 [GenBank: DQ390455.1], NR1 [GenBank: DQ364638.1] and R100 [GenBank: AP000342.1]. The β-lactam-macrolide region is identical to sequences present in plasmid pTZ3721 [GenBank: AB020531.1] and pTZ3723 [GenBank: AB038654.1]. Also of interest to us were several genes involved in siderophore production and iron metabolism. Plasmid pNRG857c has the *sitABCD *operon that encodes proteins involved in the periplasmic and inner membrane transport of iron and manganese. Two outer membrane proteins (IutA and FepA) are also encoded by the plasmid and are involved in translocation of iron across the membrane. IutA (NRG857_30235) is the ferric-aerobactin receptor, while FepA (NRG857_30015) is an iron-enterobactin outer membrane transporter, both of which are involved in the *tonB*-dependent transport pathway for iron and also the OM receptor for the colicins [33]. IutA and FepA are encoded on plasmids pAPEC-O103-ColBM, pAPEC-O1-ColBM, pCVM29188_146 (from *Salmonella enterica *serovar Kentucky, [34]), pVM01 (from the APEC strain E3, [35]), and pLVPK (from *Klebsiella pneumoniae *CG43, [36]). Interestingly, the chromosome contains a FepA paralog (NRG857_02640). The presence of several iron-acquisition genes suggests that Fur regulation of these plasmid-encoded genes occur [37,38]. As predicted, the consensus DNA sequence for Fur binding (WAATDRNWNYNAWTW) is found in the upstream regulatory region, [39]) of the *iroBCDE*, *sitABCD*, *iucABCD-iutA *operons, and the *shiF *and *fepA *genes.

### Identification of other potential virulence determinants

The chromosome of AIEC strain NRG857c encodes a variety of potential virulence factors (Table 2). As mentioned above, the plasmid carries several potential virulence factors including genes for iron acquisition. This would suggest that the plasmid contributes to the overall virulence of this bacterium, however we have demonstrated previously that a plasmid-cured variant was still able to attach to and invade epithelial cells *in vitro *[16].

**Table 2:** Putative virulence factors in NRG857c genome

Locus Tag	Gene Name	Identity (%)	Function	Related Pathotype
**Chromosome**				

***Adhesins***				
NRG857_00540	***hcpA***	89	adherence	EHEC
NRG857_05010	***csgE***	100	assembly/transport component in curli production	Common
NRG857_17655	***lpfA2***	85	major fimbrial subunit of Long Polar Fimbriae (Lpf), named *lpfA2*	EPEC, EHEC
NRG857_21765	***fimA***	77	major fimbrial subunit of type 1 fimbriae	Common
NRG857_21795	***fimH***	99	adhesin of type 1 fimbriae	Common
***Iron acquisition/Transport systems***				
NRG857_06120	***fepC***	79	ferric enterobactin transport ATP-binding protein	UPEC, EHEC
NRG857_09890	***irp2***	95	yersiniabactin biosynthetic protein	UPEC
NRG857_09895	***irp1***	91	yersiniabactin biosynthetic protein	UPEC
NRG857_09915	***fyuA***	95	pesticin/yersiniabactin receptor protein	UPEC
NRG857_17390	***chuA***	94	outer membrane receptor protein, heme utilization/transport protein	UPEC, EHEC
***Capsular and somatic antigens***				
NRG857_14650	***kpsM-II***	94	involved in polysialic acid transport, group II (K1, K4, K5, K7, K12, K92...)	
***Haemolysins and haemagglutinins***				
NRG857_06035	***clyA***	95	cytolysin, cell lysis	ETEC
NRG857_01335	***tsh***	78	temperature-sensitive hemagglutinin of avian *E. coli*, autotransporter	APEC
***Other***				
NRG857_00835	***htrA/degP***	97	stress protein, serine endoprotease	common
NRG857_02540	***ompT***	99	outer membrane protein 3b, other name: protease VII	common
NRG857_02570	***ibeB***	88	invasion gene locus (penetration of brain microvascular endothelial cells), putative resistance protein, putative outer membrane lipoprotein of copper ion antiporter	common
NRG857_04350	***ompA***	89	outer membrane protein (OMPA or OMPII)	common
NRG857_05660	***iss2***	100	gene for increased serum survival (similar to bacteriophage lambda Bor)	common
NRG857_07375	***gadB***	98	glutamate decarboxylase B, isozyme (amino acid catabolism and metabolism)	common
NRG857_11240	***ompC***	100	outer membrane protein	common
NRG857_13905	***malX***	94	maltose and glucose-specific IIABC component, pathogenicity island associated	UPEC
NRG857_15695	***nlpI***	100	lipoprotein	common
NRG857_17475	***gadA***	99	glutamate decarboxylase A, isozyme (amino acid catabolism and metabolism)	common
NRG857_19245	***dsbA***	100	oxidoreductase, thiol:disulfide interchange protein dsbA	common
NRG857_21885	***ibeA***	91	invasion protein, *E. coli *invasion of the blood-brain barrier, other name: ibe10	MENEC
***Putative virulence associated genes***				
NRG857_00565	***usp***	93	uropathogenic specific protein (putative virulence island of UPEC)	UPEC
NRG857_00950	***cadA***	68	lysine decarboxylase	common
NRG857_03880	***artJ***	92	L-arginine periplasmic binding protein, supposed to be involved in virulence	common
NRG857_05150	***mviM***	93	putative virulence factor	common
NRG857_05155	***mviN***	86	putative virulence factor	common
NRG857_05410	***b1121***	90	hypothetical protein *ycfZ*, homologous to virulence factor	common
NRG857_19995	***yjaA***	100	hypothetical protein	common
NRG857_20725	***cadA***	99	Lysine decarboxylase	common
NRG857_20730	***cadB***	100	Lysine:cadaverine antiporter	common
NRG857_22200	***nadAB***	99	meningococcal adhesion, NAD biosynthesis	common

**Plasmid**				

***Colicins and microcins***				
NRG857_30019	***cvaC***	83	structural gene for microcin V	common
NRG857_30029	***cma***	99	structural gene for colicin M	common
***Iron acquisition/Transport systems***				
NRG857_30008	***iroB***	93	siderophore	common
NRG857_30010	***iroC***	91	siderophore	common
NRG857_30012	***iroD***	99	siderophore	common
NRG857_30013	***iroE***	93	siderophore	common
NRG857_30015	***iroN***	97	siderophore	common
NRG857_30235	***iutA***	95	cloacin DF13/aerobactin outer membrane receptor protein	common
NRG857_30237	***iucD***	96	gene of the aerobactin operon, first product of the aerobactin biosynthesis pathway	common
***Other***				
NRG857_30184	***traT***	84	complement resistance protein	common
NRG857_30283	***ompT***	76	outer membrane protein 3b, other name: protease VII	common
NRG857_30309	***iss2***	100	gene for increased serum survival (similar to Bacteriophage lambda Bor)	common
***Antimicrobial resistance***				
NRG857_30085	***blaTEM***	94	ampicillin	common
NRG857_30067	***tetC***	71	tetracycline	common
NRG857_30068	***tetA***	91	tetracycline	common
NRG857_30075	***catI***	100	chloramphenicol	common
NRG857_30100	***dhfrI***	99	trimethoprim	common
NRG857_30095	***sulII***	93	sulfonamides	common
NRG857_30104	***sulI***	100	sulfonamides	common

#### (i) Type VI secretion system

We identified genes for a complete type VI secretion system (T6SS) that are associated with virulence in other invasive organisms (Table 3) [40-42]. T6SS are phage-related secretion systems found in many Gram-negative pathogens and are thought to be involved in supporting an intracellular lifestyle, although their distribution is not restricted to pathogenic bacteria [43]. The T6SS in NRG857c is found in GI-2, a low GC region of the chromosome directly downstream from a tRNA which is a common integration site for mobile genetic elements. This T6SS island encodes the conserved core elements of the secretion apparatus, including the valine-glycine repeat protein G (VgrG/NRG857_01165), the ClpV ATPase (NRG_01105) and the hemolysin coregulated protein (Hcp/NRG857_01155) that is 100% identical to Hcp in APEC-O1 and the UPEC strains UT189 and 536. We also identified a second Hcp upstream of this conserved locus (NRG_01080) that is 100% identical to Hcp in *E. coli *S88 (O45:K1:H7) that causes neonatal meningitis [44], suggesting that this T6SS island is a mosaic with different ancestries. Other organisms, including *Vibrio cholerae*, have two *hcp *genes in different parts of the genome [45], which may impart different functionalities on the secretion apparatus. Whether the T6SS in AIEC facilitates intracellular survival and/or growth will require additional experimentation that we are currently pursuing.

**Table 3:** Type VI secretion system core proteins in NRG857c

**Conserved domain(s) **^**a**^	NRG857c ortholog
ImpA N-terminal related/COG3515	NRG857_01095 hypothetical protein
IcmF-related/DUF1215/COG3523	NRG857_01090 IcmF-related protein
DUF879/COG3519	NRG857_01135 hypothetical protein
DUF877/COG3517	NRG857_01145 hypothetical protein
DUF876/COG3522	NRG857_01115 hypothetical protein
DUF770/COG3516	NRG857_01150 hypothetical protein
DUF1305/COG3520	NRG857_01130 hypothetical protein
ClpV	NRG857_01105 putative ATP-dependent Clp proteinase
FHA domain/COG3456	NRG857_01125 hypothetical protein
COG3521	NRG857_01120 hypothetical protein
DotU (IcmH)-related/COG3455	NRG857_01110 hypothetical protein
Pfam04965/COG3518	NRG857_01140 hypothetical protein
Hcp/DUF796/COG3157	NRG857_01080 hemolysin co-regulated protein
	NRG857_01155 hemolysin co-regulated protein
VgrG/DUF586/COG3501	NRG857_01165 Vgr-like protein

^**a **^as described in Reference 40

#### (ii) Adhesins

NRG857c contains genes that are important for adhesion and invasion of AIEC LF82, including *nlp1*, *htrA*, *yfgL*, and *dsbA *[46-49]. The SPAAN program [50] as well as BLASTP with relaxed stringency was used to identify and extensive list of additional predicted adhesins (Table 4). The majority of the fimbrial operons in NRG857c are found in other *E. coli *strains, with the exception of the long polar fimbriae (Lpf; NRG857-17915-17923), which might be important for tissue tropism. A second Auf fimbrial system with a potential role as a colonization factor is encoded by genes NRG857_16960 through _17005. Other potential mediators of invasion include a hemagglutinin/invasin (NRG857_17920 to _17923) and an Ibe invasin (NRG857_21885 to _21890). In previous work, the invasion of brain endothelial cells was found to be mediated by the Ibe invasin, and was located on a genomic island called GimA [51]. The presence of GimA was almost exclusive to ExPEC strains of phylogroup B2, and we now show that *ibe *is also present in AIEC, suggesting it may be involved in invasive properties of certain strains.

**Table 4:** Predicted invasion and adhesion factors in NRG857c

Locus Tag	Protein	Ortholog in:	Function	Identity (%)	SPAAN Pad-Value
***Invasion***					

NRG857_06210	putative transcriptional regulator	SMS-3-5	putative invasion gene expression up-regulator SirB	99	NA
NRG857_06250	hypothetical protein	SMS-3-5	putative invasin	93	0.44
NRG857_12485	putative intimin or invasin protein (SivH-like)	UMN026	putative intimin attaching and effacing protein or invasin protein (sivH-like)	96	NA
NRG857_13980	dinucleoside polyphosphate hydrolase	O157:H7 EDL933	putative invasion protein	100	0.12
NRG857_21885	invasion protein IbeA	SMS-3-5	invasion protein IbeA	93	0.35

***Adhesion***					

NRG857_00700	putative fimbrial-like adhesin protein	UTI89	putative fimbrial-like adhesin protein	90	0.83
NRG857_00705	protein YadK	ED1a	protein yadK, putative fimbrial-like adhesin	95	0.79
NRG857_00710	putative fimbrial-like adhesin protein YadL	S88	putative fimbrial-like adhesin protein YadL	80	0.87
NRG857_00715	putative fimbrial-like adhesin protein YadM	ED1a	putative fimbrial-like adhesin protein YadM	100	0.86
NRG857_00730	predicted fimbrial-like protein	S88	putative fimbrial-like adhesin exported protein	95	0.87
NRG857_00985	lipoprotein involved with copper homeostasis and adhesion	UTI89	lipoprotein involved with copper homeostasis and adhesion	99	0.62
NRG857_01440	putative adhesin	S88	putative adhesin	95	0.91
NRG857_01490	putative autotransporter	S88	Putative adhesin; putative outer membrane autotransporter barrel	82	0.91
NRG857_03200	hypothetical protein	K-12 substr. W3110	predicted fimbrial-like adhesin protein	95	0.26
NRG857_04950	PgaD putative PGA biosynthesis protein	K-12 substr. MG1655	required for biofilm adhesin polysaccharide PGA synthesis	88	NA
NRG857_04960	PgaB outer membrane N-deacetylase	K-12 substr. MG1655	biofilm adhesin polysaccharide PGA export lipoprotein with a polysaccharide deacetylase activity needed for export	94	NA
NRG857_04965	outer membrane protein PgaA	K-12 substr. MG1655	biofilm adhesin polysaccharide PGA export, predicted OM protein	94	NA
NRG857_05015	DNA-binding transcriptional regulator CsgD	K-12 substr. DH10B	DNA-binding transcriptional regulator of adhesion determinants	87	0.25
NRG857_05965	hypothetical protein	536	Putative adhesin	100	0.59
NRG857_06960	putative autotransported outer membrane protein involved in cell adhesion	S88	putative autotransported outer membrane protein involved in cell adhesion	78	0.96
NRG857_07415	predicted fimbrial protein-like protein	UTI89	putative fimbrial adhesin FmlD precursor	93	0.90
NRG857_07420	predicted fimbrial protein-like protein	IAI1	putative fimbrial-like adhesin exported protein	89	0.74
NRG857_07425	fimbrial-like adhesin protein	APEC O1	fimbrial-like adhesin protein	98	0.74
NRG857_08345	Hypothetical protein	SE11	putative adhesin	98	0.66
NRG857_09925	hypothetical protein	SE11	putative adhesin	95	NA
NRG857_10700	putative exported fimbrial-like adhesin protein	UTI89	putative Yeh fimbiral adhesin YehA precursor	91	0.81
NRG857_10715	putative fimbrial-like adhesin protein	ED1a	putative fimbrial-like adhesin protein	89	0.82
NRG857_10720	hypothetical protein	APEC O1	putative fimbrial-like adhesin protein	100	0.33
NRG857_11325	adhesin	O157:H7 str. EC4115	putative outer membrane autotransporter adhesin	78	0.95
NRG857_11815	putative exported fimbrial-like adhesin protein	S88	putative exported fimbrial-like adhesin protein	92	0.76
NRG857_11820	fimbrial-like protein YfcQ precursor	S88	putative fimbrial-like adhesin exported protein	99	0.83
NRG857_11825	hypothetical protein	S88	putative fimbrial-like adhesin exported protein	96	0.57
NRG857_11840	putative fimbrial-like adhesin protein	S88	fimbrial-like adhesin protein	76	0.78
NRG857_15155	putative fimbrial protein	S88	putative fimbrial-like adhesin protein	100	0.75
NRG857_15170	putative fimbrial adhesin	UTI89	putative Yqi fimbrial adhesin	95	0.84
NRG857_16975	putative fimbrial-like adhesin protein AufG	ED1a	putative fimbrial-like adhesin protein AufG	94	0.62
NRG857_17635	LpfE protein precursor	O26:H11 str. 11368	putative fimbrial adhesin protein	86	0.68
NRG857_17920	putative haemagglutinin/Invasin	CFT073	putative adhesin	87	0.98
NRG857_21795	type 1 fimbiral adhesin FimH	APEC O1	type 1 fimbrial adhesin FimH	100	0.95

In mouse models of AIEC-induced colitis, inflammation requires type I pili expression by the bacterial cells, as no colitis is induced by Δ*fimH *mutant bacteria [14]. Colitis in this model requires the expression of human CEACAM receptors by transgenic mice, suggesting that the type I pili of AIEC can induce a proinflammatory response via CEACAM receptors in the gut mucosa. In support of this, FimH, the adhesin tip protein, is necessary but not sufficient for adhesion of AIEC strain LF82 to Intestine-407 cells [52]. Polymorphisms in the FimH sequence have been identified in *E. coli *isolated from IBD patients and healthy individuals. In particular, 7 amino acid variants are associated with *E. coli *from IBD tissue and 2 variants are associated with *E. coli *from healthy individuals [53]. Interestingly, FimH in NRG857c contains two disease-associated amino acid variants (N91S, S99N, and none of the SNPs associated with healthy tissue (A48V, A140V). Whether or not these variants are associated with different inflammatory responses or subtle differences in adherence *in vivo *will be important areas for future work.

#### (iii) Transcriptional regulators of virulence genes

NRG857c contains global transcriptional regulators including *phoP-phoQ*, *envZ-ompR*, *slyA *and the negative regulators *hns*, *hha*, and *fis *involved in genome architecture and transcriptional regulation [54]. Although these transcriptional factors are common to many bacterial species, in most Gram-negative pathogens they coordinate transcription of virulence genes including secretion system, toxins, adhesins and flagellar biosynthesis machinery [55,56]. With this completed genome sequence, functional genomics approaches are now possible to understand the regulons of these transcription factors and their roles in intracellular survival and growth of AIEC. Indeed, Fis levels in the cell have already been associated with regulating the adhesive properties of AIEC strain LF82 [57].

#### (iv) Iron acquisition

**Figure 5:**
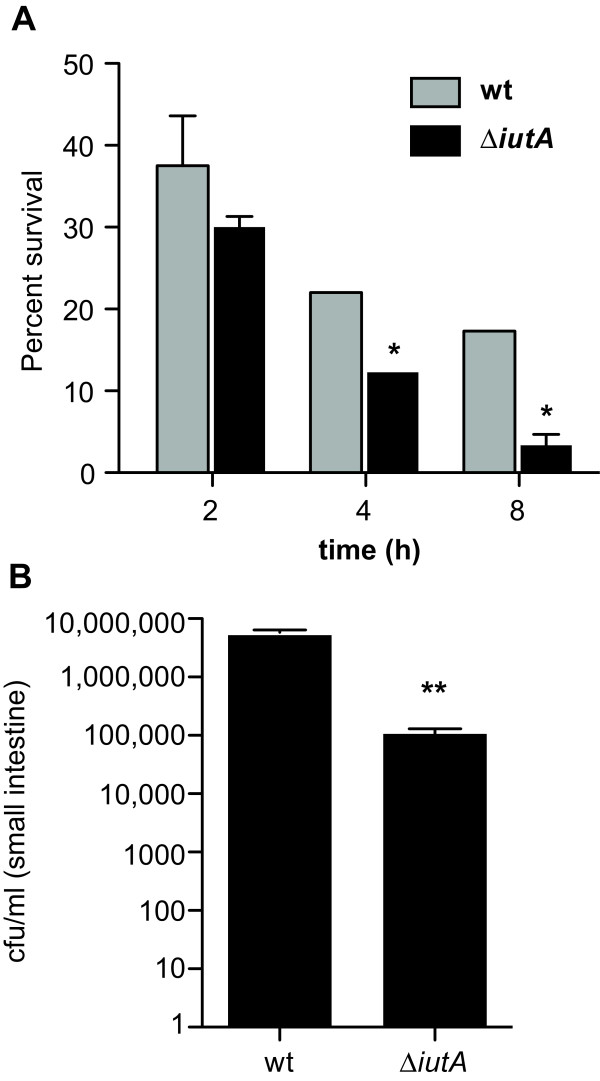
**Iron uptake by the aerobactin system is important for intracellular survival and for mouse colonization**. **(A) **J774.A1 macrophage cells were infected with wild type NRG857c or *iutA *mutant cells. The survival of intracellular bacteria was determined at various times after infection. Data are the mean survival of intracellular bacteria with standard deviation. (*, *P *< 0.05, Mann Whitney) **(B) **The aerobactin iron transport system improves colonization *in vivo*. Groups of mice were infected orally with wild type NRG857c or *iutA *mutants. Colonization of the small intestine by NRG857c AIEC was determined three days after infection by enumerating the number of cfu in tissue homogenates. Data are the means with standard errors. (**, *P *< 0.005, Mann Whitney).

Iron acquisition is an essential virulence trait in other ExPEC and these systems are expressed during urinary tract infections *in vivo *[58,59]. Since NRG875c had an abundance of iron uptake systems, we designed experiments to test the role of iron acquisition during infection. We made an aerobactin transport mutant by deletion of *iutA *and tested whether this iron transport system was important for intracellular survival and the ability to colonize animals. We found that the *iutA *mutant was able to synthesize but not transport aerobactin (Additional file 6, Table S4). To investigate the invasive properties of Δ*iutA*, we conducted standard gentamicin protection assays in J774.1 macrophage cells, which did not reveal a significant difference in the uptake at 2 h of the wild type and the *iutA *mutant (Figure 5A). However, by 4 h after infection and thereafter, the *iutA *mutant had a significant defect in intracellular survival and/or replication compared to wild type cells. To determine whether the transport of aerobactin was important for bacterial infection *in vivo*, streptomycin pre-treated mice were infected with wild type NRG857c and the isogenic *iutA *mutant as described previously for a *Salmonella *infection model [60]. Wild type NRG857c was recovered in ~50-fold more abundance in the intestinal tissue compared to Δ*iutA *(Figure 5B).

## Conclusions

The two broad hypotheses accounting for the immunopathology of IBD, including deregulation of the intestinal immune system, and dysbiosis of the commensal microbiota [61], are likely not mutually exclusive. Both pathways could be operationalized at the same time and in response to known genetic and environmental triggers. Regarding the genetic correlates of the AIEC phenotype, our genome sequence and comparative analyses provide many testable hypotheses to uncover the adhesive, invasive, and proinflammatory nature of AIEC. The fact that the 35 genomic islands in NRG857c are, in many cases, highly orthologous in LF82 but weakly conserved or absent in other *E. coli *pathotypes and commensal organisms is suggestive that these genomic islands may have an influential role in the expression of the AIEC phenotype. It is also likely that evolved differences in gene expression, or regulatory evolution, has played a pivotal role in generating phenotypic diversity involved in pathogen-like behaviour of AIEC, as we have shown previously for another intracellular pathogen [62,63]. Functional genomics studies enabled by this work will be forthcoming.

## Methods

### AIEC strain and genome sequencing

*Escherichia coli *AIEC strain NRC857c was isolated from a biopsy of a Crohn's disease patient at the Charite Hospital, Germany [16]. A mutant in aerobactin transport (designated RAA002) was created by disruption of the *iutA *gene using allelic exchange from a suicide plasmid as described previously [64]. For preparation of genomic DNA, wild type NRC857c cells were grown on solid Luria-Bertani (LB) agar at 37°C. Genomic DNA was extracted from 10 mg of bacteria scraped from a plate using the BioRobot EZ1 with the EZ1 DNA kit (Qiagen, Hilden, Germany). For plasmid purification, bacteria were grown in 4 L of LB broth and plasmid was isolated using a Maxi-prep kit (Qiagen, Hilden, Germany) according to the manufacturer's instructions. Total genomic DNA was sequenced using a Genome Sequencer FLX System (454 Life Sciences, Branford, CT, USA) at the McGill University and Genome Quebec Innovation Centre (Montreal, QC, Canada).

### Phylotype grouping, optical mapping, and *in silico *similarity clustering

Phylogenetic determinations were performed by *in silico *MLST using seven housekeeping genes (*aspC, clpX, fadD, icdA, lysP, mdh and uidA*). Analysis was performed using the software package MEGA4 [65,66] and the Neighbour-Joining method under the Tajima-Nei model. An optical map of NRG857c was generated using the restriction enzyme Nco*I *(OpGen Inc., Madison, WI) and used for contig ordering. Unweighted Pair-Group Method using Arithmetic averages (UPGMA) similarity clustering of the restriction fragments generated in the whole genome optical map of NRG857c with *in silico *maps of publicly available *E. coli *isolates was performed using MapSolver version 2.1.1 (OpGen Inc., Madison, WI).

### Gap closure

Outward facing primers annealing to adjacent contigs were designed using Primer3Plus, synthesized by SigmaGenosys (Oakville, ON, Canada) and used to amplify DNA of NRG857c using the Expand Long Template PCR system (Roche, Mannheim, Germany). PCR products were analysed on agarose gels, purified with a Montage PCR purification kit (Millipore, Billerica, MA, USA) and sequenced using Sanger sequencing (University of Guelph, ON, Canada). Finished sequence was assembled using SeqManPro (DNASTAR Inc., Madison, WI). For ribosomal RNA (rRNA) operons, primers were designed using the syntenic flanking sequences of each rRNA operon in the *E. coli *strain CFT073 [67]. These seven rDNA amplicons were sequenced using the flanking primers and specifically designed 16S (*rrs*) and 23S (*rrl*) primers based on sequence alignment with CFT073 rDNAs.

### Genome annotation and *in silico *identification of genes unique to AIEC strains, NRG957c and LF82

The genome sequence was subjected to automated annotation using the NCBI Prokaryotic Genomes Automatic Annotation Pipeline with the resulting GenBank data incorporated into Kodon (Applied Maths Inc., Austin, TX) for manual curation. A protein database was constructed from 22 *Escherichia coli *genomes available in GenBank. All of the open reading frames of NRG857c predicted by Glimmer 3 [68] were searched against the protein database using BLASTX running locally [69]. The same comparison was performed using the LF82 nucleotide sequences. A script written with the BioPerl toolkit [70] was used to parse the BLAST output files for sequences that did not have any matches, or sequences with only weak matches using the criteria: (E-value ≥ 0.01), or (Percent Identity < 50%), or (<50% of the query length was used in the BLAST alignment). The predicted ORFs of NRG857c were compared against those of strain LF82 to identify those unique to each strain. Additional comparative genomics analyses were carried out using Panseq [71] and 29 publicly-available *E. coli *genome sequences (see Additional File 7, Table S5 for list of *E. coli *genomes and accession numbers used for comparative analyses). The functions of identified sequences were predicted using the annotation engine AutoFACT [72]. Circular genome atlases were generated using CGView [73,74] or Circos [75].

### Gentamicin protection assays

J774A.1 macrophage cells were seeded at 5 × 10^5 ^cells/well in DMEM with L-glutamine and 10% FBS for 16 h prior to infection. Cells were infected at a multiplicity of infection of 10 with wild type NRG857c or the *iutA *mutant. Infected cells were incubated at 37°C for 2 h, then washed and treated for 2 h with 100 μg/ml gentamicin. At various times post-infection, cells were washed and lysed with 0.1% Triton X-100 in PBS, followed by serial plating on LB agar. Gentamicin protection experiments were performed in triplicate and reported as the percent survival with standard error with statistical significance determined by Student's t test.

### Mouse infections

All animal experiments were performed in accordance with protocols approved by the local animal ethics committee at the University of Texas Medical Branch, Galveston, Texas. Female ICR mice of 20-25-g (Charles River Laboratories) were used after 72 h of quarantine as described previously [76]. Briefly, food-restricted animals received streptomycin (5 g/L in drinking water supplemented with 7% fructose) for 48 h prior to oral inoculation with NRG857c or the *iutA *mutant. Groups of mice (n = 6) were orally inoculated with a suspension of NRG857c bacteria in a final volume of 0.4 mL delivered by gavage (20-gauge needle). The animals were maintained for 72 h, after which the animals were killed and the small intestines removed for homogenization and enumeration of the bacterial load. Groups were compared using the Mann Whitney non-parametric test.

### Siderophore utilization and iron uptake bioassays

The synthesis of siderophores by AIEC O83:H1 was analyzed by the colorimetric Arnow assay to detect catechol siderophores [77] and the ferric perchlorate assay for hydroxamates [78]. To restrict the iron availability in liquid or solid medium, the iron chelator 2,2'-dipyridil was used. To examine the ability to use various siderophores or iron compounds as iron sources, overnight cultures of AIEC O83:H1 were diluted to 1 × 10^5 ^bacteria per ml and seeded into L agar containing 2,2'-dipyridil. Plates were spotted with 5 μl of 8 μM hemin or 5 μl of an overnight culture of a siderophore-producing strain. A sterile disk containing 20 μl of 10 mM FeSO4 was placed on each plate. Growth was monitored around the spots or disk after 18 to 24 hours at 37°C.

## Authors' contributions

All authors contributed to the writing of this manuscript as well as overall project design; AK developed the gap closure strategy and manually screened the NCBI pipeline data; MM, PK and KZ carried out the laboratory experiments for closing the chromosome and plasmid sequences; AV, JN and PK performed the bioinformatics analyses.

## Supplementary Material

Additional File 1 **Alignment of Nco*I *optical map of NRC857c with nine super-contigs generated from shotgun sequencing**. The Nco*I *optical restriction map of NRG857c was aligned with the *in silico*-generated Nco*I *restriction maps of nine super-contigs arising from the shotgun sequencing and assembly of the genome. The vertical lines are alignment marks identifying similar restriction fragments between two aligned contigs. Click here for file

Additional File 2 **Accession numbers and gene coordinates used for in silico MLST analysis**. Click here for file

Additional File 3 **Alignment of Nco*I *optical map of NRC857c with the *in silico*-generated map of LF82**. The vertical lines are alignment marks identifying similar restriction fragments between two aligned contigs. The region highlighted in red is a region of DNA that is translocated in LF82. Click here for file

Additional File 4 **Table S2: Predicted Genomic Islands in NRG857c**. Click here for file

Additional File 5 **Genes unique to NRG857c and/or LF82**. Click here for file

Additional File 6 **Iron transport in AIEC NRG857c and aerobactin uptake mutant**. Click here for file

Additional File 7 **List of *E. coli *genomes used for comparative genomics analyses**. Click here for file
