# Supplementary material for: Genome sequence of adherent-invasive Escherichia coli and comparative genomic analysis with other E. coli pathotypes
Source: BMC Genomics. 2010 Nov 25;11:667. doi: 10.1186/1471-2164-11-667 (PMC3091784; doi:10.1186/1471-2164-11-667)
Supplement: Additional File 1 — Alignment of NcoI optical map of NRC857c with nine super-contigs generated from shotgun sequencing. The NcoI optical restriction map of NRG857c was aligned with the in silico-generated NcoI restriction maps of nine super-contigs arising from the shotgun sequencing and assembly of the genome. The vertical lines are alignment marks identifying similar restriction fragments between two aligned contigs. [file 1471-2164-11-667-S1.PDF]

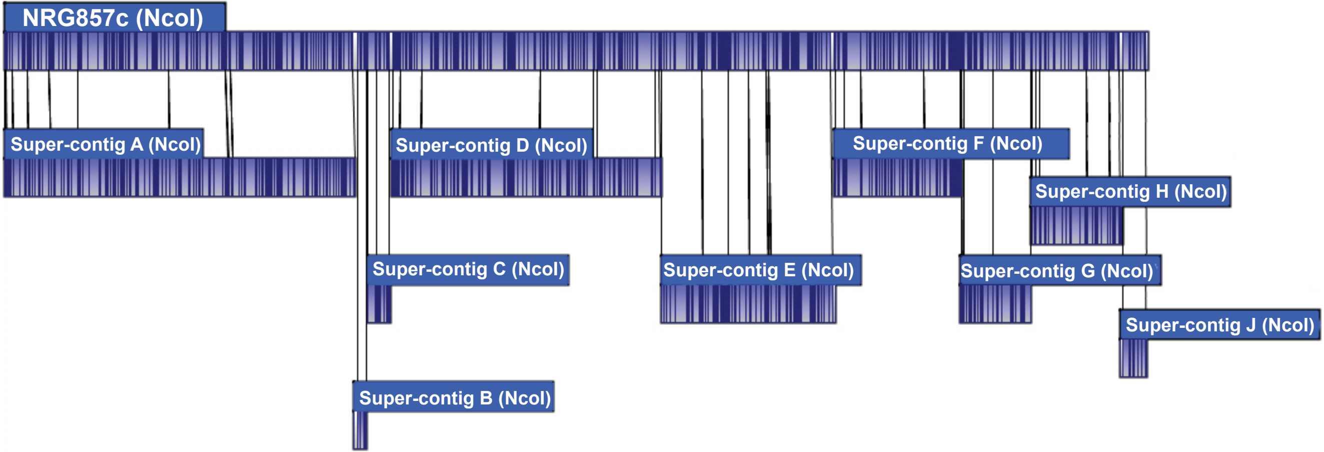
NRG857c (NcoI)
Super-contig A (NcoI)
Super-contig D (NcoI)
Super-contig F (NcoI)
Super-contig H (NcoI)
Super-contig C (NcoI)
Super-contig E (NcoI)
Super-contig G (NcoI)
Super-contig J (NcoI)
Super-contig B (NcoI)
