# Supplementary figures and images for: Genome sequence of adherent-invasive Escherichia coli and comparative genomic analysis with other E. coli pathotypes
Source: BMC Genomics. 2010 Nov 25;11:667. doi: 10.1186/1471-2164-11-667 (PMC3091784; doi:10.1186/1471-2164-11-667)

***E. coli* NRG857c (NcoI)**

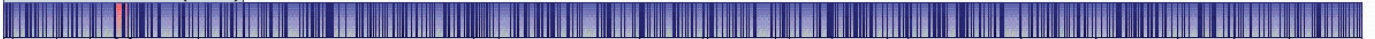

***E. coli* LF82 (NcoI) in silico**

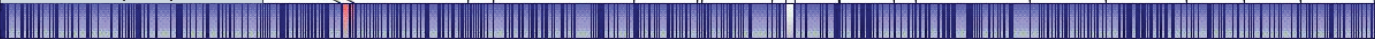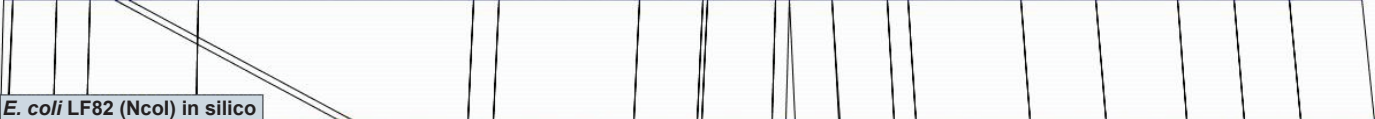

Supplement: Additional File 3 — Alignment of NcoI optical map of NRC857c with the in silico-generated map of LF82. The vertical lines are alignment marks identifying similar restriction fragments between two aligned contigs. The region highlighted in red is a region of DNA that is translocated in LF82. [file 1471-2164-11-667-S3.PDF]
