# Supplementary material for: Genome sequence of adherent-invasive Escherichia coli and comparative genomic analysis with other E. coli pathotypes
Source: BMC Genomics. 2010 Nov 25;11:667. doi: 10.1186/1471-2164-11-667 (PMC3091784; doi:10.1186/1471-2164-11-667)
Supplement: Additional File 6 — Iron transport in AIEC NRG857c and aerobactin uptake mutant. [file 1471-2164-11-667-S6.PDF]

Table S4. Iron transport in AIEC NRG857c and aerobactin uptake mutant

Strains	$FeSO_4$ Utilization [a]	Hemin Utilization [a]	Aerobactin Synthesis [b]	Aerobactin Transport	Enterobactin Synthesis	Enterobactin Transport
NRG857c	+	-	+	+	+	+
NRG857 *iutA*⁻	+	-	+	-	+	+

[a] Bioassays were performed by seeding the strain in L agar + 2,2'-dipyridil and spotting the indicated compound on the surface of the plate. + indicates a zone of growth around the iron compound. – indicates no stimulation of growth (Torres and Payne, 1997).

[b] Synthesis of siderophores was determined by chemical and bioassays as described previously (Payne, 1994).

Torres, A. G., and S. M. Payne. 1997. Haem iron-transport system in enterohaemorrhagic *Escherichia coli* O157:H7. Mol. Microbiol. **23:**825-833.

Payne SM. 1994. Detection, isolation, and characterization of siderophores. Methods Enzymol. **235:**329-44.
